# Validation of the German Classification of Diverticular Disease (VADIS)—a prospective bicentric observational study

**DOI:** 10.1007/s00384-020-03721-9

**Published:** 2020-09-04

**Authors:** Johannes C. Lauscher, Johan F. Lock, Katja Aschenbrenner, Rahel M. Strobel, Marja Leonhardt, Andrea Stroux, Benjamin Weixler, Christoph-Thomas Germer, Martin E. Kreis

**Affiliations:** 1 Department of General, Visceral and Vascular Surgery, Charité Campus Benjamin Franklin, Hindenburgdamm 30, 12203 Berlin, Germany; 2 Department of General, Visceral, Transplantation, Vascular and Pediatric Surgery, University Hospital of Würzburg, Oberdürrbacher Straße 6, 97080 Würzburg, Germany; 3 Innlandet Hospital Trust, Norwegian National Advisory Unit on Concurrent Substance Abuse and Mental Health Disorders, Brumunddal, Norway; 4 Institute of Biometry and Clinical Epidemiology, Charité – Universitätsmedizin Berlin, Freie Universität Berlin, Humboldt-Universität zu Berlin and Berlin Institute of Health, Charitéplatz 1, 10117 Berlin, Germany; 5 Berlin Institute of Health (BIH), Anna-Louisa-Karsch 2, 10178 Berlin, Germany

**Keywords:** Diverticular disease, Classification, Prospective trial, Surgical treatment, Conservative treatment, Recurrence, Quality of life

## Abstract

**Purpose:**

The German Classification of Diverticular Disease was introduced a few years ago. The aim of this study was to determine whether Classification of Diverticular Disease enables an exact stratification of different types of diverticular disease in terms of course and treatment.

**Methods:**

This was a prospective, bicentric observational trial. Patients aged ≥ 18 years with diverticular disease were prospectively included. The primary endpoint was the rate of recurrence within 2 year follow-up. Secondary outcome measures were Gastrointestinal Quality of Life Index, Quality of life measured by SF-36, frequency of gastrointestinal complaints, and postoperative complications.

**Results:**

A total of 172 patients were included. After conservative management, 40% of patients required surgery for recurrence in type 1b vs. 80% in type 2a/b (*p* = 0.04). Sixty percent of patients with type 2a (micro-abscess) were in need of surgery for recurrence vs. 100% of patients with type 2b (macro-abscess) (*p* = 0.11). Patients with type 2a reached 123 ± 15 points in the Gastrointestinal Quality of Life Index compared with 111 ± 14 in type 2b (*p* = 0.05) and higher scores in the “Mental Component Summary” scale of SF-36 (52 ± 10 vs. 43 ± 13; *p* = 0.04). Patients with recurrent diverticulitis without complications (type 3b) had less often painful constipation (30% vs. 73%; *p* = 0.006) when they were operated compared with conservative treatment.

**Conclusion:**

Differentiation into type 2a and 2b based on abscess size seems reasonable as patients with type 2b required surgery while patients with type 2a may be treated conservatively. Sigmoid colectomy in patients with type 3b seems to have gastrointestinal complaints during long-term follow-up.

**Trial registration:**

https://www.drks.de ID: DRKS00005576

## Introduction

The prevalence of diverticulosis and diverticular disease (DD) is rising in the western population. It is associated with increasing age—approximately 5% of the population under the age of 40 suffer from diverticulosis or DD and up to 65% of people aged 65 or more [1]. Patients with diverticulosis suffer from acute diverticulitis in 10–25% in the course of their lifetime. Another 15–20% with acute diverticulitis develop complications such as perforation, abscess, fistula, or stenosis [2].

In the last three decades, many classifications of DD were introduced, namely Hinchey [3] and modified Hinchey [4]. The internationally used modified Hinchey classification provides a detailed analysis of perforated diverticulitis irrespective of the abscess size and does not include uncomplicated and chronic recurrent types of diverticular disease [4].

These classifications have their limitations. The Classification of Diverticular Disease (CDD) as a more complex classification was introduced in Germany in 2014 (Table 1) [5–7]. CDD differentiates between acute uncomplicated phlegmonous diverticulitis without abscess (type 1b), and different types of acute complicated DD—covered perforation with micro-abscess ≤ 1 cm (type 2a), covered perforation with macro-abscess > 1 cm (type 2b), and free perforation (type 2c). According to the German CDD guideline, type 2a may be treated conservatively with antibiotics and does not require surgery in most cases, and type 2b is normally treated with antibiotics, percutaneous drainage when feasible, and elective sigmoid colectomy [6]. The CDD lists three types of chronic DD (relapsing or persistent symptomatic diverticular disease)—namely type 3a as symptomatic uncomplicated DD, type 3b as relapsing diverticulitis without complications, and type 3c with complications such as colonic stenoses, fistulas, or inflammatory mass.

**Table 1:** Classification of Diverticular Disease (CDD) [6]

Type 0	*Asymptomatic diverticulosis*	Random finding; asymptomatic
Type 1	*Acute uncomplicated diverticular disease/diverticulitis*	
Type 1a	Diverticulitis/diverticular disease without peridiverticulitis	Symptoms attributable to diverticula Signs of inflammation (lab tests): optional Typical cross-sectional imaging
Type 1b	Diverticulitis with phlegmonous peridiverticulitis	Signs of inflammation (lab tests): mandatory Cross-sectional imaging: phlegmonous peridiverticulitis
Type 2	*Acute complicated diverticulitis* As 1b, plus:	
Type 2a	Micro-abscess	Covered perforation, small abscess (≤ 1 cm); minimal paracolic air
Type 2b	Macro-abscess	Paracolic or mesocolic abscess (> 1 cm)
Type 2c	Free perforation	Free perforation, free air/fluid Generalized peritonitis
Type 2c1	Purulent peritonitis	
Type 2c2	Fecal peritonitis	
Type 3	*Chronic diverticular disease* Relapsing or persistent symptomatic diverticular disease	
Type 3a	Symptomatic uncomplicated diverticular disease	Typical clinical features Signs of inflammation (lab tests): optional
Type 3b	Relapsing diverticulitis without complications	Signs of inflammation (lab tests): present Cross-sectional imaging: typical
Type 3c	Relapsing diverticulitis with complications	Identification of stenoses, fistulas, conglomerate tumors
Type 4	*Diverticular bleeding*	Identification of source of bleeding

The hypothesis of the prospective bicentered observational VADIS trial (*Va*lidation of the Classification of *Di*verticular Di**s**ease) was that CDD classifies diverticulitis with phlegmonous peridiverticulitis correctly as acute uncomplicated DD in terms of need for surgery, rate of recurrence, and long-term quality of life in comparison with acute complicated DD with micro-abscess and macro-abscess. We hypothesized that type 2a and type 2b differ regarding the short-term and long-term courses and, in addition, that relapsing diverticulitis without complications and with complications has different short-term and long-term outcomes. Accordingly, we hypothesized that types 1b and 2a do not generally benefit from sigmoid colectomy, while type 2b has a better outcome after surgery.

## Method

### Trial oversight

VADIS was a bicentric, prospective observational trial. Patients with proven DD were prospectively included and follow-up was conducted 1 and 2 years after inclusion.

The study protocol was approved by the Ethics Committee of the Charité – University Medicine Berlin (Application No. EA4/092/13). The trial was conducted in accordance with the ethical principles of the Declaration of Helsinki and the principles of Good Clinical Practice (ICH-GCP E6) [8]. The VADIS trial is registered at “Deutsches Register Klinischer Studien” https://www.drks.de (ID: DRKS00005576) and reported according to STROBE statement [9].

### Patients and therapeutic strategy

**Fig. 1:**
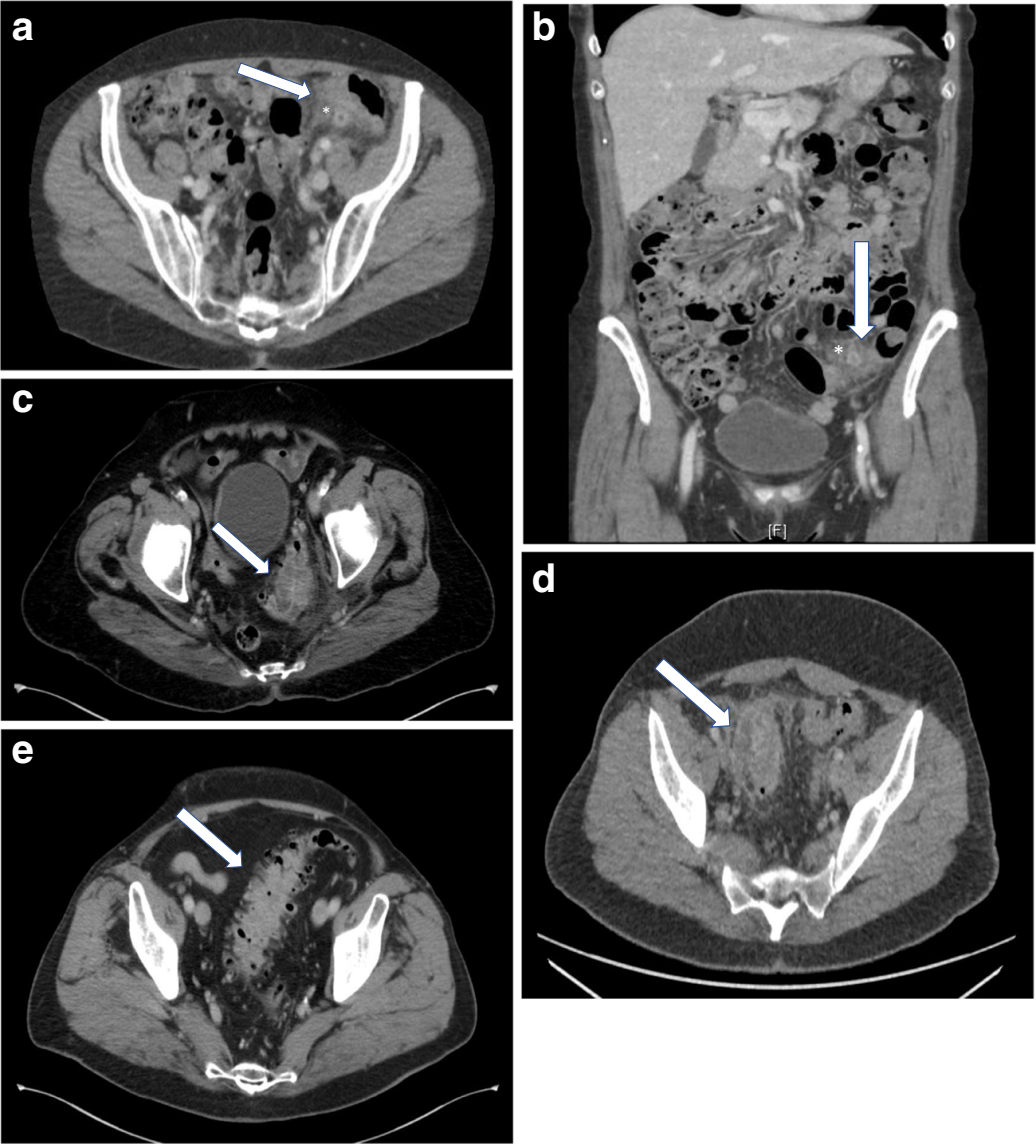
CT images illustrating different types of diverticular disease according to CDD. CDD type 1b in axial (**a**) and coronal (**b**) view. Asterisks mark inflamed diverticula. White arrows show phlegmonous inflammation of pericolic tissue (fat tissue stranding). **c** CDD type 2a with phlegmonous inflammation of pericolic tissue plus micro-abscess (0.9 cm) marked by a white arrow as a sign of covered perforation. **d** CDD type 2b with phlegmonous inflammation of pericolic tissue plus pericolic macro-abscess (5 × 1.5 cm) marked by a white arrow as a sign of covered perforation. **e** CDD type 3b with radiologic signs of chronic recurrent inflammation with thickening of the colonic wall, luminal narrowing, and multiple non-irritated diverticula (marked by white arrow)

Patients 18 years or older, capable to give informed consent, and suffering from diverticular disease (DD) were eligible to participate. Types 3a (symptomatic uncomplicated diverticular disease) and type 4 (diverticular bleeding) were excluded because these are distinct disease entities. DD was proven clinically (abdominal pain AND increase in white blood cell count > 11/nl OR increase in C-reactive protein > 5 mg/dl) and confirmed in spiral computerized abdominal tomography scan. The diagnostic criteria in cross-sectional imaging were direct proof of inflamed diverticula, bowel wall thickening > 3 mm, increased contrast enhancement, perifocal mesenteric injection, or free abdominal fluid according to recent guidelines for DD [5, 7]. All patients were classified according to CDD (Table 1). For representative images of different types of diverticular disease, see Fig. 1a–e.

Patients were enrolled by one of the surgeons involved in the trial. All patients were clinically examined and a blood sample was taken. Health-related quality of life was assessed at the time of inclusion using the validated questionnaires Gastrointestinal Quality of Life Index (GIQLI) [10] and Short Form 36 Health Survey (SF-36) [11].

**Fig. 2:**
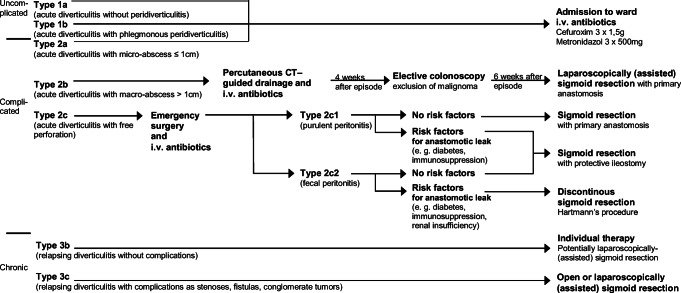
Treatment of diverticular disease according to CDD [5, 7]

Patients were treated according to the German CDD (Fig. 2) [5–7]. Conservative treatment was defined as admission to ward, intravenous antibiotic treatment, and, if necessary, insertion of percutaneous abscess drainage by interventional radiology. A percutaneous abscess drainage was inserted in patients with macro-abscess when technically feasible. Antibiotic treatment included cefuroxime (M.P.I. Pharmaceutica, Hamburg, Germany)—three times per day 1.5 g intravenously—and metronidazole (Braun, Bethlehem, USA)—three times per day 500 mg intravenously. Initial/primary surgical treatment was defined as sigmoid resection including the upper third of the rectum either during the initial stay or within 8 weeks after the first admission. According to the CDD guideline, the initial surgical treatment was recommended in type 2b (acute diverticulitis with macro-abscess), type 2c (free perforation), and type 3c (chronic recurrent diverticulitis with complications). Types 1b and 2a were only operated initially if patients suffered from persistent symptoms. In patients with type 3b (chronic recurrent diverticulitis without complications), an individual decision was made depending on the frequency and severity of diverticulitis episodes, comorbidities, and age. Sigmoid colectomy was performed in a standardized fashion. The period of hospitalization including readmission due to postoperative complications was documented 30 days postoperatively.

### Outcomes

The primary endpoint was the recurrence of DD within a 2-year follow-up. The previously described criteria for DD were again required for the diagnosis of recurrence. Treatment of a recurrent episode was assessed as regards outpatient treatment, admission to hospital, and conservative or surgical treatment with potential postoperative complications.

Secondary endpoints were as follows:

1. Frequency of gastrointestinal symptoms after 2 years. Persistent abdominal pain, bloating, and painful constipation were measured by a questionnaire with 5-point Likert scale.
2. Gastrointestinal Quality of Life Index (GIQLI) after 2 years with an evaluation of the total score of the questionnaire as described previously.
3. Health-related quality of life measured by the Short Form 36 Health Survey (SF-36) questionnaire after 2 years.
4. Postoperative complications during the primary stay and early elective interval or surgery due to the recurrence of DD. Relaparotomy was defined as an unplanned laparotomy due to a postoperative complication within 30 days after sigmoid resection.

Data were collected on paper-based case report forms at the time of recruitment (visit 1), directly after surgery during the primary stay and early elective interval (visit 2), 30 days after the operation (visit 3), 1 year (visit 4), and 2 years (visit 5) after the episode of DD leading to inclusion in VADIS. Follow-up after 1 and 2 years was conducted by telephone interview.

### Statistical analysis

The primary endpoint recurrence was analyzed with cross-tabulation and chi-square test. Frequency of gastrointestinal symptoms, subgroup analysis of CDD and association between recurrence of DD and health-related quality of life after 2 years, and primary treatment were also assessed by cross-tabulation and chi-square test. For quantitative outcomes such as the secondary parameters GIQLI and SF-36, statistical group comparisons were performed using the *t* test for independent variables. Additional parameters were depicted according to their scale and distribution with absolute and relative frequencies for categorical parameters and mean and standard deviation for quantitative parameters. *p* values ≤ 0.05 were considered statistically significant. Statistical analysis was carried out using IBM SPSS Statistics 25® (IBM, Armonk, NY, USA).

## Results

### Patient characteristics

**Fig. 3:**
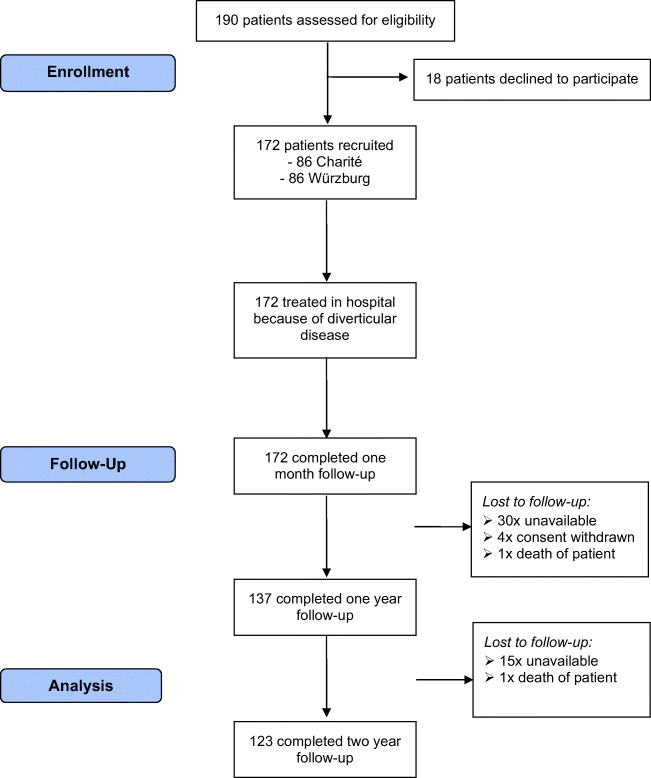
Flow diagram of VADIS study

Between November 2013 and September 2015, 190 patients were assessed for eligibility. Eighteen patients declined to participate, and 172 patients were recruited in the two participating centers, 86 respectively. One hundred twenty-three patients (72%) could be analyzed in the follow-up 2 years after inclusion. The flow diagram of the VADIS is given in Fig. 3. Table 2 shows the baseline characteristics of the patient cohort.

**Table 2:** Baseline characteristics of patient cohort

	Patient cohort (*n* = 172)
Sex	
Female	83 (48.3%)
Male	89 (51.7%)
Age (years; mean ± SD)	61.0 ± 13.1
BMI (kg/m^2^; mean ± SD)	28.2 ± 7.0
Current treatment	
Anticoagulants	34 (19.8%)
Glucocorticoids	17 (9.9%)
Other immunosuppressive medication	3 (1.7%)
Radiotherapy within 6 weeks	2 (1.2%)
Chemotherapy within 6 weeks	3 (1.7%)
Current smoking	38 (22.1%)
Alcohol abuse	5 (2.9%)
Comorbidities	
Coronary artery disease	31 (18.0%)
Liver cirrhosis	2 (1.2%)
Chronic obstructive pulmonary disease	11 (6.4%)
Diabetes mellitus	17 (9.9%)
Malignant disease (current or h/o)	15 (8.7%)
Number of episodes of DD in total (mean ± SD)	3.1 ± 4.8
Patients with first episode of DD	74 (43.0%)
CRP at time of admission (mg/dl; mean ± SD)	42.8 ± 62.5
CRP ≥ 50 mg/dl at time of admission	42 (24.4%)
White cell count at time of admission (/nl; mean ± SD)	11.9 ± 8.4
White cell count > 11/nl or < 4/nl at time of admission	83 (47.2%)
Clinical examination at presentation	
Local peritonism	61 (35.5%)
Generalized peritonism	16 (9.3%)
Abdominal tenderness without peritonism	95 (55.2%)
Classification of diverticular disease	
Type 1a	4 (2.3%)
Type 1b	56 (31.8%)
Type 2a	21 (11.9%)
Type 2b	18 (10.2%)
Type 2c1	7 (4.0%)
Type 2c2	0 (0%)
Type 3b	54 (30.7%)
Type 3c	12 (6.8%)
GIQLI at inclusion (mean ± SD)	97.3 ± 21.8

Data are *n* (%) or mean ± SD; Other immunosuppressive medications including methotrexate, azathioprine, and biologicals; alcohol abuse > 15 standard drinks/week. *SD* standard deviation; *BMI* body mass index; *h/o* History of; *DD* diverticular disease; *CRP* C-reactive protein; *GIQLI* Gastrointestinal Quality of Life Index.

### Comparison type 1b vs. type 2a/b

**Fig. 4:**
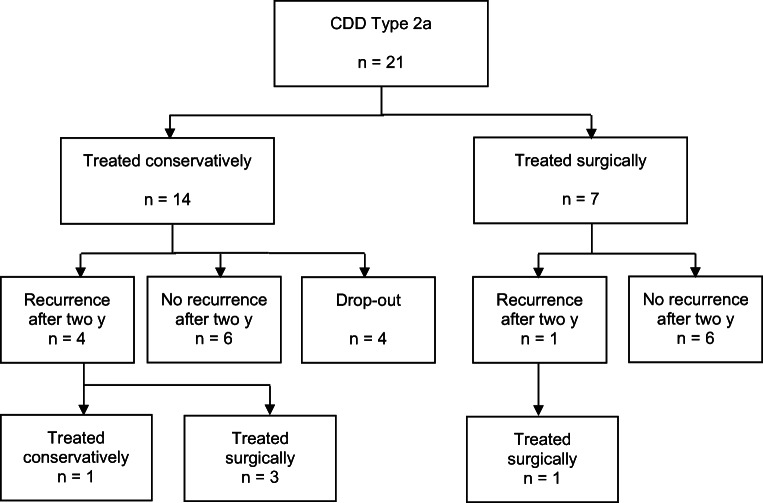
Treatment of patients with CDD type 2a after 2 years

**Fig. 5:**
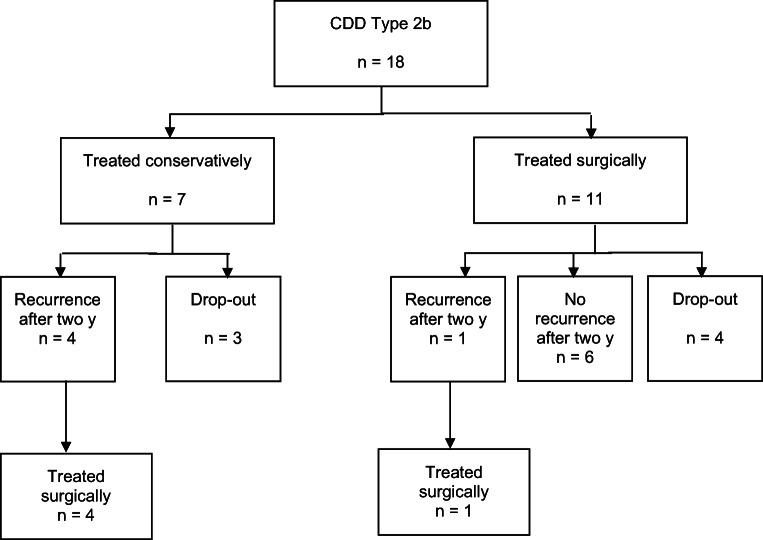
Treatment of patients with CDD type 2b after 2 years

Patients with acute uncomplicated diverticulitis with phlegmonous peridiverticulitis (type 1b) required less often surgery during the primary stay and early elective interval than patients with acute complicated diverticulitis with micro-abscess (type 2a) or macro-abscess (type 2b): 3 (5%) vs. 18 (46%) patients (*p* < 0.001). Twenty patients (46%) with type 1b had recurrent DD within 2 years and ten patients (36%) with type 2a/b (*p* = 0.41). Eight of 20 patients with type 1b and initial conservative treatment had to be operated for recurrence (40%) vs. eight/ten patients with type 2a/b (80%) (*p* = 0.04) (Table 3, Figs. 4 and 5).

**Table 3:** Comparison between CDD type 1b and type 2a/b

	Type 1b (*n* = 56)	Type 2a/b (*n* = 39)	*p* value
Surgery during primary stay or within eight weeks after first admission (early elective)	3 (5.4%)	18 (46.2%)	< 0.001┴*
Length of hospital stay including readmission to ward (days)	5.6 ± 4.6	11.3 ± 7.6	< 0.001▪*
Follow-up after 2 years	44	28	
Recurrence of diverticular disease	20 (45.5%)	10 (35.7%)	0.41┴
Surgery due to recurrence	8/20 (40.0%)	8/10 (80.0%)	0.04┴*
Admission to ward due to recurrence	16/20 (80.0%)	8/10 (80.0%)	1.00┴
Abdominal pain after 2 years			0.51┴
Never	27 (61.4%)	19 (67.9%)	
Rare	7 (15.9%)	6 (21.4%)	
Sometimes	8 (18.2%)	3 (10.7%)	
Permanent	2 (4.5%)	0 (0.0%)	
Bloating after 2 years			0.51┴
Never	21 (47.7%)	10 (35.7%)	
Rare	7 (15.9%)	9 (32.1%)	
Sometimes	12 (27.3%)	7 (25.0%)	
Permanent	4 (9.1%)	2 (7.1%)	
	Type 1b (*n* = 44)	Type 2a/b (n = 28)	*p* value
Painful constipation after two years			0.53┴
Never	32 (72.7%)	16 (57.1%)	
Rare	6 (13.6%)	6 (21.4%)	
Sometimes	4 (9.1%)	3 (10.7%)	
Permanent	2 (4.5%)	3 (10.7%)	
GIQLI at inclusion	101 ± 20	100 ± 20	0.77▪
GIQLI after 2 years	121 ± 16	118 ± 16	0.50▪
SF-36/MCS after 2 years	52 ± 10	49 ± 12	0.19▪
SF-36/PCS after 2 years	46 ± 10	47 ± 12	0.79▪

Data are *n* (%) or mean ± SD; *CDD* Classification of Diverticular Disease; *SD* standard deviation; *GIQLI* Gastrointestinal Quality of Life Index; *SF-36* Short Form 36 Health Survey; *MCS* mental component summary; *PCS* physical component summary. ^┴^Chi-square test; *p ≤ 0.05; ▪*T* test for independent variables

No difference between type 1b and type 2a/b was found in terms of long-term health-related quality of life (SF-36), long-term GIQLI, and long-term gastrointestinal symptoms (Table 3).

### Comparison type 2a vs. type 2b

Patients with type 2a showed a trend to require less surgical treatment during primary stay than patients with type 2b: seven (33%) vs. eleven (61%) (*p* = 0.08). No difference in the rate of recurrence within 2 years was found between type 2a and 2b. Three out of five patients with type 2a (60%) and five out of five patients with type 2b (100%) with recurrent DD needed surgery (*p* = 0.11). All five patients with type 2b who were initially treated conservatively needed colonic resection due to recurrence (Figs. 4 and 5).

Patients with type 2a had a higher GIQLI-score after 2 years (123 ± 15) than patients with type 2b (111 ± 14) (*p* = 0.05). Patients with type 2a performed better in the MCS scale of SF-36 than those with type 2b: 52 ± 10 vs. 43 ± 13 (*p* = 0.04) (Table 4).

**Table 4:** Comparison between CDD type 2a and type 2b

	Type 2a (*n* = 21)	Type 2b (n = 18)	*p* value
Surgery during primary stay or within eight weeks after first admission (early elective)	7 (33.3%)	11 (61.1%)	0.08┴
Length of hospital stay including readmission to ward (days)	8.5 ± 4.7	14.6 ± 9.1	0.01▪*
Follow-up after 2 years	17	11	
Recurrence of diverticular disease	5 (29.4%)	5 (45.5%)	0.39┴
Surgery due to recurrence	3/5 (60.0%)	5/5 (100.0%)	0.11┴
Admission to ward due to recurrence	3/5 (60.0%)	5/5 (100.0%)	0.11┴
Abdominal pain after 2 years			0.12┴
Never	14 (82.4%)	5 (45.5%)	
Rare	2 (11.8%)	4 (36.4%)	
Sometimes	1 (5.9%)	2 (18.2%)	
Permanent	0 (0.0%)	0 (0.0%)	
GIQLI at inclusion	102 ± 18	99 ± 22	0.63▪
GIQLI after 2 years	123 ± 15	111 ± 14	0.05▪
SF-36/MCS after 2 years	52 ± 10	43 ± 13	0.04▪*
SF-36/PCS after 2 years	46 ± 12	49 ± 10	0.53▪

Data are *n* (%) or mean ± SD; *CDD* Classification of Diverticular Disease; *SD* standard deviation; *GIQLI* Gastrointestinal Quality of Life Index; *SF-36* Short Form 36 Health Survey; *MCS* mental component summary; *PCS* physical component summary. ^┴^Chi-square test; *p ≤ 0.05; ▪*T* test for independent variable

### Comparison type 3b vs. type 3c

**Fig. 6:**
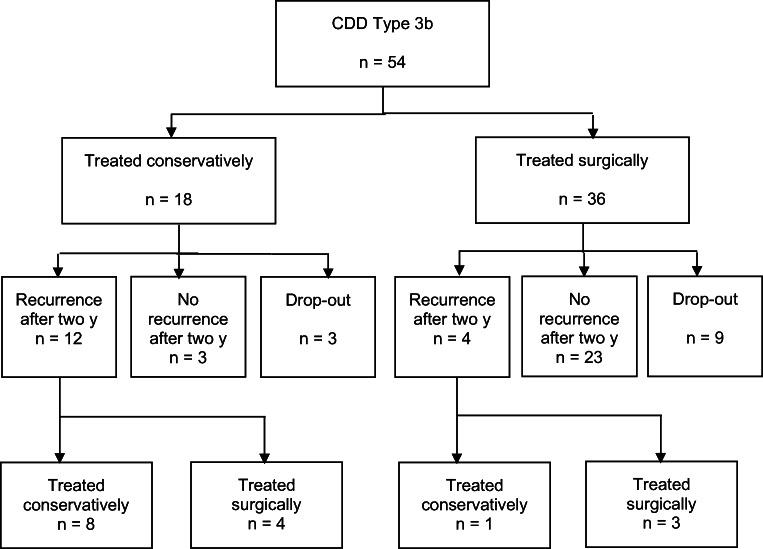
Treatment of patients with CDD type 3b after 2 years

Patients with recurrent diverticulitis without complications (type 3b) required less often surgery during primary stay than patients with relapsing diverticulitis with complications (type 3c): 36 (67%) vs. 12 (100%) (*p* = 0.02). The rate of relaparotomy was 8% in type 3b vs. 25% in 3c (*p* = 0.31). Seventeen (41%) patients with type 3b suffered from recurrence of DD, while eight were treated with sigmoid colectomy (Fig. 6). None of the patients with type 3c had relapse of DD.

Patients with type 3b tended to have lower scores in GIQLI after 2 years than those with type 3c: 110 ± 21 vs. 128 ± 9 (*p* = 0.07). Patients classified as type 3b scored less in the “General Health” scale of SF-36 than those classified as type 3c: 60 ± 22 vs. 83 ± 8 (*p* = 0.04). The same applied to the “Mental Health” scale of the SF-36: 70 ± 20 vs. 91 ± 11 (*p* = 0.04) (Table 5).

**Table 5:** Comparison between CDD type 3b and type 3c

	Type 3b (*n* = 54)	Type 3c (*n* = 12)	*p* value
Surgery during primary stay or within eight weeks after first admission (early elective)	36 (66.7%)	12 (100.0%)	0.02┴*
Length of hospital stay including readmission to ward (days)	10.6 ± 6.8	19.3 ± 10.1	0.008▪*
Follow-up after 2 years	42	4	
Recurrence of diverticular disease	16 (38.1%)	0 (0.0%)	0.13┴
Surgery due to recurrence	7/16 (43.8%)	0/0 (0.0%)	0.38┴
Admission to ward due to recurrence	11/16 (68.9%)	0 (0.0%)	0.23┴
Abdominal pain after 2 years			0.12┴
Never	22 (51.2%)	4 (100.0%)	
Rare	8 (18.6%)	0 (0.0%)	
Sometimes	7 (16.3%)	0 (0.0%)	
Permanent	6 (14.0%)	0 (0.0%)	
GIQLI at inclusion	87 ± 21	100 ± 22	0.08▪
GIQLI after 2 years	110 ± 21	128 ± 9	0.07▪
SF-36/MCS after 2 years	48 ± 11	58 ± 4	0.08▪
SF-36/PCS after 2 years	48 ± 10	54 ± 2	0.004▪*
SF-36/general health after 2 years	60 ± 22	83 ± 8	0.04▪*
SF-36/mental health after 2 years	70 ± 20	91 ± 11	0.04▪*

Data are *n* (%) or mean ± SD; *CDD* Classification of Diverticular Disease; *SD* standard deviation; *GIQLI* Gastrointestinal Quality of Life Index; *SF-36* Short Form 36 Health Survey; *MCS* mental component summary; *PCS* Physical component summary. ^┴^Chi-square test; *p ≤ 0.05; ▪*T* test for independent variable

### Comparison of surgical and conservative treatment in CDD

There was neither a difference between the surgical and conservative treatment of patients with type 2a in terms of recurrence of DD, GIQLI-scores, gastrointestinal symptoms after 2 years, nor long-term quality of life in SF-36 (Table 6).

**Table 6:** Subgroup analysis of CDD and association between quality of life after 2 years and primary treatment

CDD	Follow-up after 2 years	Treated primarily surgically (*n* = 44)	Treated primarily conservatively (*n* = 70)	*p* value
Type 2a	Total patients (*n*)	7	10	0.24
	GIQLI	128 ± 10	119 ± 17	0.24
	SF-36/physical functioning	88 ± 22	69 ± 32	0.18
	SF-36/social functioning	93 ± 19	85 ± 18	0.39
	Abdominal pain	1 (14.3%)	2 (20.0%)	0.76
	Bloating	4 (57.1%)	5 (50.0%)	0.77
	Painful constipation	3 (42.9%)	4 (40.0%)	0.91
Type 2b	Total patients (*n*)	7	4	
	GIQLI	115 ± 13	104 ± 16	0.23
	SF-36/physical functioning	83 ± 22	64 ± 43	0.34
	SF-36/social functioning	91 ± 12	47 ± 21	0.002*
	Abdominal pain	3 (42.9%)	3 (75.0%)	0.30
	Bloating	5 (71.4%)	4 (100.0%)	0.24
	Painful constipation	3 (42.9%)	2 (50.0%)	0.82
Type 3b1.1.1.1.1.1.	Total patients (*n*)	27	15	
	GIQLI	110 ± 21	107 ± 24	0.65
	SF-36/physical functioning	80 ± 21	77 ± 26	0.64
	SF-36/social functioning	84 ± 20	81 ± 25	0.63
	Abdominal pain	10 (37.0%)	10 (66.7%)	0.07
	Bloating	18 (66.7%)	15 (100.0%)	0.01*
	Painful constipation	8 (29.6%)	11 (73.3%)	0.006*

Data are mean ± standard deviation or *n* (%); *CDD* Classification of Diverticular Disease; *GIQLI* Gastrointestinal Quality of Life Index; *SF-36* Short Form 36 Health Survey; *p ≤ 0.05. **▪***T* test for independent variables

Surgically treated patients with macro-abscess (type 2b) reached higher scores in the “Social Functioning” scale of the SF-36 after 2 years than conservatively treated patients: 91 ± 12 vs. 47 ± 21 (*p* = 0.002) (Table 6).

Fewer patients with type 3b who underwent sigmoid colectomy suffered from bloating (67% vs. 100%; *p* = 0.01) and painful constipation (30% vs. 73%; *p* = 0.006) than those with conservative treatment. Patients after surgery trended to have less abdominal pain after 2 years (37% vs. 67%; *p* = 0.06) (Table 6).

### Operative procedure and postoperative complications

Laparoscopic sigmoid resection with primary anastomosis was done in 41 cases, laparoscopic converted to open sigmoid resection with primary anastomosis was done in 13 cases, open sigmoid resection with primary anastomosis was performed in 43 cases, and open Hartmann resection in three cases. There was no 30-day postoperative mortality in all types of CDD. The rate of anastomotic leakage was seven/79 (9%) in primarily operated patients and in none out of 23 patients who were operated with recurrent DD. Severe perioperative complications (Clavien-Dindo ≥ 3) occurred in twelve/79 (15%) patients who were operated primarily and in none out of 23 (0%) patients who were operated due to recurrent disease.

## Discussion and conclusions

Despite the high prevalence of DD, a uniform international classification and convincing evidence about the optimal treatment strategy are missing to date. As there are different types including acute uncomplicated and complicated DD and chronic recurrent DD, the recent German guideline for DD aims to give a comprehensive classification system and therapeutic outline [5–7].

This was the first prospective study to evaluate the German CDD. We could show that the majority of patients with acute phlegmonous diverticulitis without abscess (type 1b) can be treated conservatively without adverse short-term and long-term outcomes. It seems justified to classify these patients as uncomplicated. The VADIS study detected differences between patients with micro-abscess (type 2a) and macro-abscess (type 2b). While all patients with type 2b required surgery, the majority of patients with micro-abscess could be treated conservatively and were not in need of colectomy during follow-up. What is more, the long-term quality of life of patients with macro-abscess was worse. As far as chronic recurrent diverticulitis is concerned, patients with type 3b had a favorable long-term course with less gastrointestinal complaints after surgery compared with conservative management.

The recent guidelines for DD recommend conservative treatment of type 1b without sigmoid resection in the disease-free interval [5]. In the VADIS study, patients with type 1b required primary surgery in less than 5%, whereas patients with types 2a/b did more often (46%). Patients with type 1b needed less often surgery in the long-term course due to recurrence than types 2a/b: 40% vs. 80%. In both groups—type 1b and types 2a/b—the number of patients with recurrent DD within 2 years was high (46% and 36%). In a retrospective cohort analysis, 48% of patients suffered from recurrence after conservative treatment, of whom 86% were classified to stage Hinchey I—mild cases of diverticulitis such as phlegmon or small abscess [12]. The rate of recurrence may depend on the severity of DD [5]. Retrospective data is heterogenous with rates of nearly 2% after uncomplicated diverticulitis [13] and 35% after complicated DD; of these, 16% were subsequently operated [14].

According to VADIS, in type 1b, gastrointestinal symptoms did not differ regardless of whether patients had been operated during primary stay or not. Brandlhuber et al. found in their retrospective study that patients with type 1b had lower scores on the GIQLI when they were operated, more pain in the lower left abdomen, and more frequent diverticulosis-associated complaints after surgery [15]. Holmer et al. showed that the conservative treatment of phlegmonous diverticulitis led to a nearly complete regression of inflammation in histology [16].

Another novelty in CDD is the differentiation between type 2a and type 2b. All patients with type 2b who were not operated primarily developed recurrence which needed to be operated. Patients with type 2b showed a trend in having worse long-term GIQLI. In addition, their mental health score in SF-36 was worse compared with type 2a. Brandlhuber et al. showed that patients with type 2a had more diverticulosis-associated symptoms and worse health-related quality of life when they were operated [15]. VADIS trial did not find any difference between the conservative and surgical treatment of type 2a. Patients with micro-abscess may therefore be treated conservatively. Surgically treated patients with macro-abscess showed better “Social Functioning” in SF-36. Taken together, VADIS provides evidence that patients with type 2b DD may be operated initially in contrast to patients with type 2a. It appears useful to differentiate acute diverticulitis according to abscess size because these types differ in terms of treatment and outcome. This is in accordance with the findings of Brandlhuber et al. After being operated, patients with type 2b suffered less from pain in the lower left abdomen and less frequently from diverticulosis-associated symptoms than those without operation [15].

Another new aspect of CDD is the subdivision of chronic recurrent DD (types 3a/b/c). VADIS showed that the differentiation between type 3b and type 3c is reasonable. Recurrent diverticulitis with complications is a more severe disease and surgery is more demanding than in type 3b. Twenty-five percent of patients with type 3c underwent a relaparotomy. On the other hand, there was a trend towards patients with type 3c having higher scores in GIQLI after 2 years compared with type 3b. Patients with type 3c assessed their general state of health and their mental health better after 2-year follow-up. Evidence is provided that types 3b and 3c are indeed different entities of DD. Whereas type 3c seems to have a longer and more complicated postoperative course, type 3b appears to have a more unfavorable long-term course with more recurrences, more gastrointestinal symptoms, and worse quality of life.

Interestingly, patients with type 3b benefitted from sigmoid colectomy. Surgically treated patients had less bloating and less painful constipation after 2 years. These results are confirmed in other studies. A multicenter randomized trial revealed that patients with recurrent or ongoing abdominal complaints after an episode of diverticulitis benefitted from an elective sigmoid resection compared to conservative therapy, resulting in a higher GIQLI at 6 months’ follow-up [17]. In a retrospective study, 89% of patients with chronic uncomplicated diverticulitis—requiring a minimum of 3 months of clinical symptoms or radiographic signs—benefitted from surgery with acceptable morbidity rates [18].

According to the German guideline for DD, type 3b should be operated only after a careful assessment of risks and benefits depending on the clinical symptoms [5, 6]. In view of the results of VADIS, the indication for sigmoid resection in patients with type 3b may be extended and patients should be informed that colon resection may be associated with less long-term gastrointestinal symptoms.

Several potential limitations of the trial must be taken into account. First, it was an observational trial without randomization. All patients were prospectively recruited with regular and close follow-up. Second, recurrences of DD were not again classified according to CDD. The severity of recurrent DD could be estimated by the treatment of recurrent disease. The same criteria for DD were again required for the diagnosis of recurrence. Since not all patients were hospitalized for recurrence and treated in one of the two study centers, the rate of recurrence may have been overestimated. The somewhat high rate of recurrences after surgical treatment of DD might also be caused by this phenomenon.

The VADIS study provides evidence that the German Classification of Diverticular Disease (CDD) is feasible and allows differentiation into types of diverticulitis which leads to appropriate treatment. Surgery for recurrence was not associated with more complications than primary surgery. Other trials confirmed that recurrences do not imply higher risk of complications compared with the first episode [12, 19].

CDD classifies type 1b correctly as uncomplicated diverticulitis which should be treated conservatively. The differentiation in micro-abscess and macro-abscess is justified as patients with macro-abscess require surgery while patients with micro-abscess may be treated conservatively in the majority of cases with success. The long-term quality of life of patients with macro-abscess was worse compared with patients with micro-abscess. Considering recurrences and long-term quality of life, patients with macro-abscess benefitted from primary elective sigmoid colectomy. Evidence was provided that patients with relapsing diverticulitis without complications may benefit from elective sigmoid colectomy which appears to reduce long-term gastrointestinal symptoms.

## Data Availability

All original data and material is available and can be provided to the journal on request.
